# Correction: Outcome of acute bacterial meningitis among children in Kandahar, Afghanistan: A prospective observational cohort study

**DOI:** 10.1371/journal.pone.0293150

**Published:** 2023-10-12

**Authors:** Bilal Ahmad Rahim, Niamatullah Ishaq, Ghulam Mohayuddin Mudaser, Walter R. Taylor

There are errors in the Funding section. The correct Funding statement is: This study did not receive any specific funding. WR Taylor is part funded by Wellcome under grant 220211. For the purpose of Open Access, the author has applied a CC BY public copyright licence to any Author Accepted Manuscript version arising from this submission.
