# Supplementary figures and images for: Outcome of acute bacterial meningitis among children in Kandahar, Afghanistan: A prospective observational cohort study
Source: PLoS One. 2022 Apr 11;17(4):e0265487. doi: 10.1371/journal.pone.0265487 (PMC9000062; doi:10.1371/journal.pone.0265487)

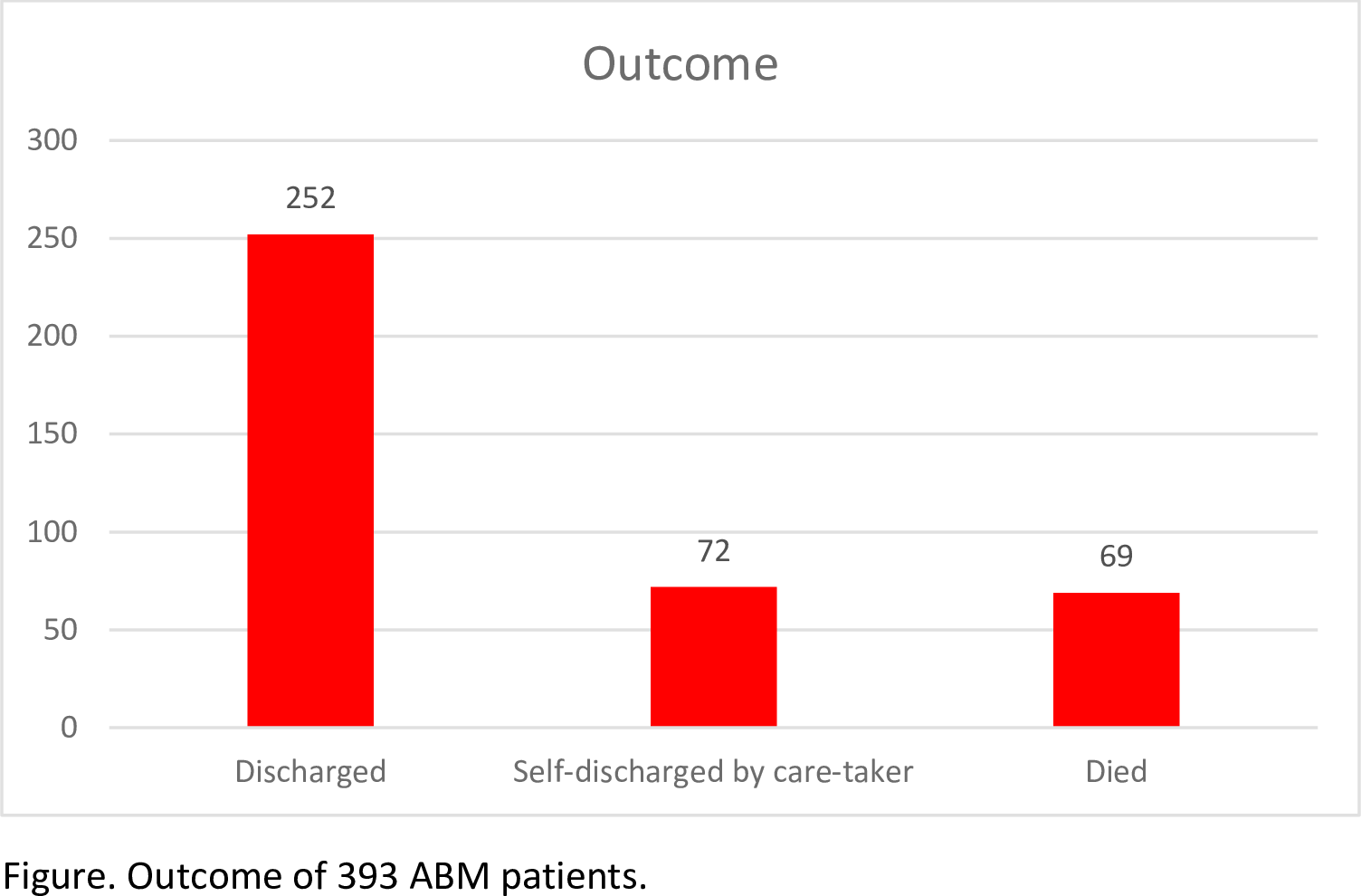

Supplement: S1 Fig — (TIF) [file pone.0265487.s001.tif]
