# Supplementary material for: Outcome of acute bacterial meningitis among children in Kandahar, Afghanistan: A prospective observational cohort study
Source: PLoS One. 2022 Apr 11;17(4):e0265487. doi: 10.1371/journal.pone.0265487 (PMC9000062; doi:10.1371/journal.pone.0265487)
Supplement: Attachment — Submitted filename: Response to Reviewers.docx [file pone.0265487.s003.docx]

Hello dear academic Editor,

Many thanks for your prompt reply and useful comments. These are really important points.

Following are your comments and our answers:

**Comment 1.** The first and most important is that a definition of acute bacterial meningitis must be provided.

**Answer:** We had written the definition at the end of “Material and Methods”. Our definition is based on WHO guidelines. Now we gave it a heading, and is as follows:

**Definition of ABM**

ABM was defined according to the guidelines of WHO, i.e., a CSF examination showing at least one of the following characteristics: turbid CSF appearance, leukocytosis >100 cells/mm^3^, and leukocytosis of 10–100 cells/mm^3^ with either an elevated protein level (>100 mg/dl) or decreased glucose level (<40 mg/dl) [14].

**Comment 2.** Second, there is a description of CSF findings but only with a range given that includes WBCs as low as 5 and neutrophil counts as low as 0 or lymphocytes up to 100. What is the reason for believing that these were bacterial (or were not TB)?

**Answer:** We have selected and recruited all the ABM patients based on CSF analysis results from the laboratory. Lab results were analyzed based on the definition by WHO, as mentioned in the answer of first comment. We recruited the patient if he/she had **any** **one** of the following 3 findings in CSF analysis:

- Turbid CSF appearance.
- Leukocytosis >100 cells/mm^3^.
- Leukocytosis of 10–100 cells/mm^3^ with either an elevated protein level (>100 mg/dl) or decreased glucose level (<40 mg/dl).

**Comment 3.** One another note, the sample size calculation appears to be irrelevant to the study being reported.

**Answer:** Yes, we agree with you. It is irrelevant here. So, we have corrected the sample size calculation in the manuscript as follows:

**Sample size calculations**

All eligible patients, who were admitted to pediatric ward of Mirwais regional hospital during 12-month-period (February 2020–January 2021) and willing to take part in the study, were included in this study. So, there was no need for the sample size calculation.
