# Supplementary material for: Outcome of acute bacterial meningitis among children in Kandahar, Afghanistan: A prospective observational cohort study
Source: PLoS One. 2022 Apr 11;17(4):e0265487. doi: 10.1371/journal.pone.0265487 (PMC9000062; doi:10.1371/journal.pone.0265487)
Supplement: Attachment — Submitted filename: Response to Reviewers.docx [file pone.0265487.s004.docx]

First of all, I am very thankful of the useful comments of the two reviewers. I really appreciate it. As we have very less facilities (especially in research) in war-torn Afghanistan, we have tried our best to show the world a real picture of pediatric acute bacterial meningitis in Kandahar, Afghanistan. But I agree that there were many mistakes in our manuscript. Thanks again for your kind and fruitful comments.

6. Review Comments to the Author

Please use the space provided to explain your answers to the questions above. You may also include additional comments for the author, including concerns about dual publication, research ethics, or publication ethics. (Please upload your review as an attachment if it exceeds 20,000 characters)

Reviewer #1: Abstract
Line 25: Background – no need to say handicap as this is covered by the term morbidity

I removed it based on your comment.

Introduction
- Reference number 3 is not useful to this paper as it is from 1993 and treatment and outcomes have improved much since then

I removed the old reference. Now the references are up-to-date.

- Line 59: remove “three most common bacteria”

I removed it.

- Lines 59 – 61: the information included from reference no. 4 is outdated and has been replaced with a newer Lancet study (GBD 2016 Meningitis Collaborators. Global, regional, and national burden of meningitis, 1990-2016: a systematic analysis for the Global Burden of Disease Study 2016. Lancet Neurol. 2018 Dec;17(12):1061-1082)

Thanks for the link. I removed old data. Now the data is replaced with the reference that you had mentioned.

- Consider including some data from the region (as local data is not available)

I added the data of Afghanistan and its surrounding neighbor countries.

Methodology
- Lines 83 – 85: need to indicate which data was collected from the questionnaire and to whom were these questionnaires administered. Also, indicate which data was abstracted from patient files.

I added the following details:

Data was collected during 12-month-period (February 2020–January 2021) using questionnaire with questions regarding socio-demographic characteristics, physical signs and symptoms, laboratory examinations, and treatment of the patients admitted with the diagnosis of ABM. All the patients were followed up during the whole period of their hospitalization. All the questionnaires were filled by the study investigator. For all the patients, detailed history and physical examinations was conducted by experienced pediatricians while laboratory investigations and treatment data were abstracted from the patient files.

- How was vaccination status assessed?

I added these details:

Vaccination status of the younger children were assessed by looking at the vaccination cards (or recall if the card was left at home) while for the older children by recall of the parents or caretakers. If the caretakers did not have more and accurate information, other family members of the patients were contacted via phone calls better and accurate data collection.

- Line 95: define what treatment outcomes your study is looking at

To assess the treatment outcome (survived or died) of ABM among children in Kandahar province, Afghanistan.

- Line 96: describe the factors that you will include in assessing this objective

To determine the risk factors (including socio-demographic status, physical signs and symptoms, laboratory examinations, and treatment) associated with death among ABM patients.

- Line 99: who made the diagnosis of ABM and how was it made i.e. primary clinician Or study investigator? If made by clinician please provide some detail on how the clinicians in the facility make this diagnosis? is made in this facility). If by study investigator, explain at what point and how this was done.

Patient admitted in pediatric ward with the diagnosis of ABM (diagnosis was made by expert pediatricians of the pediatric ward, based on WHO guidelines, i.e., a CSF examination showing at least one of the following characteristics: turbid CSF appearance, leukocytosis >100 cells/mm3, and leukocytosis of 10–100 cells/mm3 with either an elevated protein level (>100 mg/dl) or decreased glucose level (<40 mg/dl) [14]).

- Lines 103 & 104: how were the diagnoses of TBM and encephalitis made and confirmed; Note that CSF findings from table 5 suggest that patients might have had TBM and viral meningitis/encephalitis

•	Tuberculous meningitis patients (Clinical entry criteria plus a total diagnostic score of ≥10 points (when cerebral imaging is not available) or ≥12 points (when cerebral imaging is available) plus, exclusion of alternative diagnoses. At least 2 points should either come from CSF or cerebral imaging criteria) [15].

•	Viral meningitis patients (patients with clinical diagnosis of viral meningitis and CSF analysis showing lymphocytic predominance [>100 cells/mm3], protein <45 mg/dl, and glucose >40 mg/dl).

- Line 105: why were patients with a history of parenteral broad-spectrum antibiotics use before admission excluded from the study? Also define which antibiotics were included in this category.

I added these lines:

Children who received any parenteral broad-spectrum antibiotics before admission (as these antibiotics may interfere with the results of CSF analysis). The two groups of broad-spectrum antibiotics used and available in Kandahar are third generation cephalosporins and macrolides.

- Line 110: instead of sample size calculation, power calculation can be done and they can be used to demonstrate the minimum detectable difference with different sample sizes.

The second reviewer also commented on the sample size calculation. He commented to write “Not applicable” in it. So, I did that. Hope you also agree with that.

- Line 128 – 133: The definition of ABM – not clear why this has been included – this should be covered under line 99 and has been addressed above.

OK. I addressed it under line 99, as per your comment.

Results:
- Consider providing the flow of study participants – i.e. how many patients were excluded by each criteria indicated in lines 103 – 106

Based on your comment, I added the following information in the result section:

Among the excluded patients, 158 patients had TB meningitis, 121 had encephalitis, 27 had received intravenous ceftriaxone before admission, while parents of 19 patients refused to participate in the study.

- The results of variables with outliers should include median (IQR) e.g. age, duration, CSF cell count, differentials etc.

I added median (IQR) for all the variables with outliers.

- The overall presentation of results in tables 1 and 6 should be done in a logical and more meaningful manner. Consider reducing the number of tables and assign variables to the appropriate table i.e.
o Table 1 : baseline demographic and social characteristics including vaccination data (can have both categorical and continuous data)
o Table 2: clinical presentation
o Table 3: lab findings including CSF findings
o Table 4: treatment and outcome findings

Based on your comment, I reduced the number of tables from 6 to 4. Now all the relevant data is in its appropriate table, both categorical and continuous.

- There is no need to include the total row for each variable under all tables (a single N at the top should suffice) and also there is no need to include the total number in the write-up of the results, just the number and percent suffices e.g. no need for 138/339 (58.8%) for male gender.

OK. I removed the total row from the variables in all the tables. A single (e.g., N=393) has been added at the top titles row. Also I removed the total number in the results.

- De-clutter the tables – there are too many variables being presented and not all are necessary; consider dropping e.g. number in the same room, no. of <5 in the same room, age of starting breastfeeding, birth order, twins, mode of delivery etc

I removed all the unnecessary variables from the tables.

- Dexamethasone therapy, Duration of admission (LOS), no of days of iv ceftriaxone and no. of days of iv ampicillin should be in the treatment & outcome table (not in table 1)

I transferred the data to the treatment and outcome table.

- Also consider presenting antibiotic data as one variable i.e. duration of anti-meningitic antibiotics.

OK. I changed them to one variable, i.e., duration of anti-meningitis antibiotics.

- Some variables presented in the results are not clear and should be defined either in the methodology section or as a footnote in the tables e.g. income categories, breastfeeding, bottle feeding;

OK. I defined them.

- The symptoms and signs variables should also be defined in terms of, at what point were they obtained i.e. at admission, or at any point in their stay.

OK. These symptoms were at the time of admission.

- Age categories – consider having an additional category for above 12 years

OK. I added another category of >12 years of age.

- Weight and height should be presented as wt for age and ht for age
- Vaccination variables: Why have the HiB and PCV vaccinations been combined into a single variable?

I had combined the Hib and PCV vaccines because they both are administered at the same age and time (on week 6, 10 and 14).

But now as per your comment, I separated them as 2 separate variables.

- Comorbidity variable: This variable needs to be defined and categorized as either acute or chronic; the acute conditions are most likely concurrent infections; need to reconsider how this data has been presented. Moreover, the acute concurrent infections should be in the clinical presentations table while the chronic underlying co-morbidities should be in the baseline characteristics table

OK. I corrected this variable. Now its name is changed to “concurrent infections”. Also, I put it in clinical presentation table.

- Table with lab results should include units e.g. TLC, platelets, WBC in CSF

OK. Now they all have the units, e.g., cells/mm^3^.

- Results write-up should include the most relevant information from the tables and to the overall objective e.g.
o Lines 156-158 should include something on next stiffness or headaches
o Lines 162-163: should include information on CSF white cell count and differential results

OK. I added the data of the variables that you mentioned.

- Consider excluding the patients that were self-discharged from the treatment & outcome table as they could not be assessed for complications and outcomes.

OK. Thanks. It was really a big mistake. Now we corrected it. Self-discharged patients are now removed from the outcome analysis. I re-do all the analysis based on you comment.

- Table 7:
o Current table 7 analysis is testing for factors associated with survival not mortality: redo this table and assess for mortality across all variables and should indicate the reference categories against each variable, as this has also not been done in a uniform manner

I did the analysis again in all the variables. I also added the reference categories.

o The values presented for the OR and p-value for living in over-crowded areas is incorrect, needs to be redone; also reconsider using this variable as 99.2% of participants were living in the crowded areas (>5).

OK. I removed the variable of “living in over-crowded areas” from the analysis.

o This table does not need the total number in each row as well (total N at the top is good enough).

OK. I removed all the “total” rows from each variable and put N at the top.

o De-clutter this table similar to the suggestion for table 1 and 2 above.

OK. I did it.

o Consider not testing for complications as a factor associated with mortality. Most complications assessed in this study are likely assessed at discharge or at recovery. Also, death can be considered as a complication of ABM.

OK. Thanks. It was really a mistake. I removed the variable of “complications” from the analysis.

o Assess acute and chronic co-morbidities separately

OK. I changed it as per your previous comment and changed it to “concurrent infections”.

Discussion
- Case Fatality Rate – exclude the number who were self-discharged.

OK. I removed the self-discharge patients from this.

- The references given for the proportions of case fatality and complication from the various countries should also include the populations in which these studies were done as they are not all the same e.g. some are in infants, others in children under 5 and others across all pediatric age groups.

OK. It is done. Now I included the information of study participants with data of each country.

- Lines 240 – Should read: …will have incremental effects in reducing the global burden…

OK. I corrected and added it.

- As mentioned above, living in crowded areas has been incorrectly included.

OK. I have removed the living in over-crowded areas from the analysis.

Conclusion:
- Line 274: incorrect conclusion: your study does not prove the cost-effectiveness of vaccination.

It was a mistake. Now we corrected it.

- Lines 275-276: not sure how the assessment of outcomes and factors was hindered by unavailability of culture confirmed cases.

OK. We removed this sentence. Our aim by this sentence was the unavailability of definite diagnosis.

Reviewer #2: August 28, 2021

PLOS ONE
Manuscript ID: PONE-D-21-20289R1
Title: Treatment outcome of acute bacterial meningitis among children in Kandahar, Afghanistan: A prospective study

Thank you for the opportunity to review this interesting manuscript. The authors conducted a prospective observational cohort study of 393 children <18 years of age with acute bacterial meningitis (ABM) at Mirwais Regional Hospital in Afghanistan from February 2020 through January 2021. The sociodemographic components reflected many patients coming from low-income families living in overcrowded households in rural areas and with incomplete childhood vaccination programmes. The in-hospital mortality was 17.6% and adjusted analyses suggested not having received vaccination for Hib or PCV, living in overcrowded houses, complications during admission, and lack of adjunctive dexamethasone as poor prognostic factors.

The authors should be commended for performing an important study during very difficult and challenging circumstances. The scientific community definitely needs more data from developing countries. However, the methodology can still be improved in several ways which I believe would strengthen the impact and credibility of the data substantially.

Major concerns:
1. Abstract. Conclusion. Although there is no doubt that Hib and PCV vaccinations prevent ABM, the provided data do not document a protective effect of Hib or PCV vaccinations on incidence of ABM in children in Afghanistan. This would require a comparison of risk of ABM among vaccinated and unvaccinated children. So, the conclusion that their findings prove the value does not seem to be justified in this dataset. However, they can/should still encourage more widespread implementation of the vaccinations, which makes sense. Please comment.

Thanks for the comment. Now I corrected the sentence with encouraging more widespread implementation of the Hib and PCV vaccinations.

2. It is my overall impression from the provided methods and the discussion, that they examine in-hospital mortality as primary outcome, which should be clearly specified throughout the abstract and manuscript. Of note, this is not the same as ‘case-fatality rate’ which is also used once in the discussion. Please correct if I understand your study correctly.

OK. It was our mistake. Now it is changed to “in-hospital mortality”.

3. ‘Comorbidities’ usually denote pre-existing conditions such as diabetes mellitus, arterial hypertension etc. Suggest changing to ‘predisposing’ or ‘concomitant’ infection throughout the manuscript.

OK. This comment was given by the other respectable reviewer also. I corrected it and changed to “concurrent infections”.

4. The authors conducted a prospective observational cohort study. How were the data retrieved, personal interview or medical record review?

All the questionnaires were filled by the study investigators. For all the patients, detailed history and physical examinations was conducted by experienced pediatricians while laboratory investigations and treatment data were abstracted from the patient files.

5. There are no missing values at all, which seems almost impossible. Especially since some patients were up to 15 years old and the parents may not remember for how long the child was breastfed, length of bottle-feeding etc (which is also likely to be irrelevant in adolescents). Another example is presence of headache which is 100% complete but many cases were babies who are very unlikely to be able to account for presence of headache or not. Were families and relatives contacted to complete all variables and what if they couldn’t be reached? Please comment.

Yes, we agree with you. It is very difficult not to have missing data. Although we have very limited resources here in Afghanistan, but I am very fortunate to have a very committed team. They tried a lot not to miss any information. But I am sure that there might be recall bias in the answers of mothers or caretakers of the children. For breastfeeding, it was information of exclusive breastfeeding (only breastfeeding during first 6 months of life). For headache, we included we had included the very young children as “No headache”. Now we corrected it by putting headache as “not applicable” or “missing” data.

Vaccination status of the younger children were assessed by looking at the vaccination cards (or recall if the card was left at home) while for the older children by recall of the parents or caretakers. If the caretakers did not have more and accurate information, other family members of the patients were contacted via phone calls better and accurate data collection.

6. Please specify how you categorized socio-economic status and income into e.g. low, middle, and high-income families?

We had put it based on monthly income of the family. Now we changed SES to “monthly income”.

7. Please describe who retrieved the study data or performed the interviews (single or multiple authors)?

All the questionnaires were filled by the study investigators (we had 3 data collectors). For all the patients, detailed history and physical examinations was conducted by experienced pediatricians while laboratory investigations and treatment data were abstracted from the patient files.

8. Table 4. Is it symptoms and signs at admission or in total during course of illness? The proportion with seizures seems extremely high (95%). History of seizures or observed? Misclassification? Please comment?

The signs and symptoms were on admission. Now we added “on admission” with clinical features. Regarding seizures, it was the presence of seizures in the last 48 hours before admission.

9. Co-infections and complications, e.g. in Table 6. Some patients were likely to have had several co-infections (e.g. otitis media and pneumonia) or complications (seizures and cerebral palsy). Please account for this in the data provided.

Yes, you are absolutely right. We should have added this data. Now we added the data of patients who has more than one concurrent infections or complications.

10. Table 7. Please show the reference used for each crude (and adjusted) odds ratio, i.e. male sex in analyses of sex as prognostic factor etc.

OK. Now we added the references.

Minor suggestions:
1. Title. Suggest adding ‘cohort study’ to ‘a prospective study’

OK. Now “A prospective observational cohort study” is added to the title.

2. Abstract. Suggest specifying ‘complications’.

OK. Now we specified the main complications in the abstract.

3. Abstract. The results suggest ‘not administering Hib and PCV vaccines’ was a poor prognostic factor. Would it not be more accurate to write ‘no Hib or PCV vaccines’ since I assume that they refer to children receiving childhood vaccinations before falling ill?

OK. We corrected it as ‘no Hib or PCV vaccines’ both in abstract in the main text.

4. Please clarify in the methods which diagnostic methods were available in your setting, e.g. microscopy of CSF but no culture etc.

OK. Now we clarified this important point in the manuscript.

5. Suggest to simply put in ‘Not applicable’ under sample size calculations.

OK. I made it as per your comment.

6. Line 141-143 is a bit unclear to me. Please clarify.

OK. Now the sentence is made clear.

7. Please define ‘coma’ in your study.

OK. Now definition of comma is added. “Coma is defined as a state of deep unconsciousness from which the affected person cannot be aroused even by strong stimulation.”

8. Suggest to also include differences in study populations and definitions of ABM as a reason for differences in mortality across studies.

OK. Now we have added them.

9. Line 177 onwards you should delete “%” after the 95% confidence interval and the p-value does not add any information, e.g.: “crude odds ratio [COR] 0.1, 95% CI 0.0-0.1%, and p-value <0.001).
10. The crude odds ratio for coma for in-hospital mortality is currently 0.1 (95% CI 0.0-0.1), which is clearly reversed?

OK. % and p-values have been removed from these places. We did the analysis again. You were right. Now COR of coma is 3.7.

11. Please also check that you have the right reference level in adjusted odds ratios in Table 8, e.g. for adjunctive dexamethasone currently reads as an AOR of 6.2 for in-hospital mortality?

OK. We again did the analysis of all the variables.

12. Line 234-237. The sentences are a bit unclear with different numbers?

OK. Those 2 sentences were unclear. Now it is OK and clear.

13. Line 242. Overcrowded houses are likely a risk factor for contracting ABM, but your study examines the prognosis after falling ill with ABM. However, it may be a proxy for poverty and a general poor health and thereby an adverse prognostic factor?

OK. The other reviewer had also mentioned this issue and suggest to remove it as nearly all (99.2%) of participants were living in the over-crowded areas. So I removed this variable.

14. Line 275. This should be moved to limitations instead of in the conclusions.

OK. I moved this sentence to the limitations of the study.

Best of luck to the authors.

Many thanks for your kind comments.
