# Supplementary material for: Outcome of acute bacterial meningitis among children in Kandahar, Afghanistan: A prospective observational cohort study
Source: PLoS One. 2022 Apr 11;17(4):e0265487. doi: 10.1371/journal.pone.0265487 (PMC9000062; doi:10.1371/journal.pone.0265487)
Supplement: Attachment — Submitted filename: Response to Reviewers.docx [file pone.0265487.s005.docx]

First of all, I am very thankful of the useful comments of the reviewer. I really appreciate it. As we have very less facilities (especially in research) in war-torn Afghanistan, we have tried our best to show the world a real picture of pediatric acute bacterial meningitis in Kandahar, Afghanistan. But I agree that there were many limitations in our study. Thanks again for your kind and fruitful comments.

6. Review Comments to the Author

Please use the space provided to explain your answers to the questions above. You may also include additional comments for the author, including concerns about dual publication, research ethics, or publication ethics. (Please upload your review as an attachment if it exceeds 20,000 characters)

Reviewer #1: Overall ,the authors have done a great job in improving their manuscript. They must be commended for taking their time in addressing each and every single issue that had been raised and have done so with great detail.

Nevertheless, there are still some issues that need to be addressed and these are listed below:

1. Line 1 - Consider removing "Treatment" from the title: the study's primary objective was to report on mortality and not treatment.

Answer: OK. Removed.

2. Line 25 - drop "still".

Answer: OK. Dropped.

2. Line 33 - drop "by".

Answer: OK. Dropped.

3. Line 44 - drop "not giving".

Answer: OK. Dropped.

4. Line 45 - drop "no" and combine HiB and PCV vaccine. (This is according to your analysis since you assumed both Hib and PCV were given but this is a significant flaw in your study - refer to comment 11 below)

Answer: OK. Done.

5. Lines 49 - 50 - Conclusion should be based on your study findings while acknowledging limitaitons. Your study cannot conclude that "ministry of public health should increae coverage". Consider rewording this statement along the lines of "...increasing vaccine coverage can potentially reduce the risk of death from ABM in children in Afghanistan..."

Answer: OK. Done. Conclusion sentence has been changed.

6. Lines 62 - 68: move these sentences ("In 2016.." to "respectively[5]") to the next paragraph.

Answer: OK. Done.

7. Line 81: Add "The" before "Afghanistan".

Answer: OK. Done.

8. Line 85: Add "The" before "main".

Answer: OK. Done.

9. Lines 92-93: Author needs to specify which variables were obtained from interviewing parents/guardians and which ones were obtained by abstraction from clinical records.

Answer: OK. Done as follows: Data of the patients admitted with the diagnosis of ABM werewas collected during 12-month-period (February 2020–January 2021) using questionnaire with questions variables regarding socio-demographic characteristics, and physical signs and symptoms obtained from physical examination or interviewing the parent/guardian of the patients; while , laboratory examinations, and treatment variables extracted from clinical records of the patients.

10. Lines 96-98: AS mentioned above, indicate which of the above variables were based on physician documentation in clinical records.

Answer: OK. Done.

11. Lines 102-103: How did authors determine whether patients had received both Hib and PCV vaccines? Even though given at the same age, it is possible that some might have missed one or the other. Also, note according to lines 82-83 PCV-13 was introduced 4 years after Hib, therefore it is not correct to assume that because a child received a vaccine at 6, 10 or 14 weeks they received the Hib and PCV-13. Also, can you please explain how you accounted for those who did not complete vaccination series e.g. if they only received 1 or 2 doses.

Answer: Yes, we totally agree with your kind comment. Our main confirmation of the Hib and PCV vaccines were based on seeing the EPI card of the child. If card was not available, our decision was based on the recall of the parents or guardians, which we agree that can be biased.

We tried our best to be accurate as possible, but we admit that we could not avoid some of the main problems, like re-call bias.

12. Line 107: Add "were" before willing.

Answer: OK. Added.

13. Lines 111-112: Drop these lines; not required in a manuscript

Answer: OK. Dropped.

14. Line 114: Drop "treatment"

Answer: OK. Dropped.

15. Line 117: Consider dropping "risk"

Answer: OK. Dropped.

16. Line 118: Add "children with" before "ABM" and drop "patients".

Answer: OK. Added.

17. Lines 120-124: Is the WHO criteria used by said pediatricians in their routine practice or were they instructed to apply these during study period?

Answer: Yes, the pediatricians were routinely using the WHO criteria.

18. Line 121: consider dropping "expert"

Answer: OK. Dropped.

19. Line 125: Drop "Both male and female"

Answer: OK. Dropped.

20. Lines 128-131: Need to explain who made this diagnosis. Was it the attending paediatrician? or was this determined by study investigators? Also what is the name of this score? Is this score used in routine practice or was this specifically used during the study period?

Answer: OK. Explained. This score of diagnosis is routinely used by pediatricians in the hospital. First on admission diagnosis is made by the pediatricians, then our investigators were re-doing it for the inclusion in our study.

21. Lines 132-1314 Need to explain who made this diagnosis. Was it the attending paediatrician? or was this determined by study investigators? Also is the criteria used in routine practice or was this specifically used during the study period?

Answer: OK. Explained. This score of diagnosis is routinely used by pediatricians in the hospital. First on admission diagnosis is made by the pediatricians, then our investigators were re-doing it for the inclusion in our study.

22. Line 138: It is not clear why macrolides were excluded from this study? Macrolides are unlikely to have an effect on CNS infections.

Answer: Yes, we agree. It is corrected to “Penicillins”.

23. Lines 170-171: Move the sentence on adjunctive dexa therapy to the paragraph of table 4 i.e. Lines 197-201

Answer: OK. Done .

24. Line 176 (Table 1): Move the row of age(mean) to the row with the age categories. Also present the median(IQR) for age as it is unlikely you have a normal distribution.

Answer: OK. Done.

25. Line 185 (Table 2): Headache - in the methods section please explain why the N for headaches is less than the total. Explain that it was limited to those whom headache could be assessed.

Answer: OK. Done.

26. Line 185 (Table 2): No. of concurrent infections - consider dropping this row. This does not add much value to the study and it is extremely unusual to have two or more true infections besides meningitis.

Answer: Ok. Done.

27. Line 186: Coma should be defined using clinical criteria i.e. based on GCS or AVPU scale

Answer: OK. Done.

28. Line 194 (Table 3): What was the N for this table?

Answer: It is 393. Now added.

29. Line 197-199: Move the lines of concurrent infections to the write up of Table 1 (from lines 161-172)

Answer: OK. Done. It belongs to write up of table 2, so I moved it to Table 2.

30. Line 203 (Table 4): Exclude the self-discharged group entirely from this table. All the variables in this table are dependent on the patient being admitted and therefore cannot be included here. The exclusion of self-discharged group from this part of the analysis should be stated in the methodology.

Answer: OK. Done. All the table is re-made based on your comments.

31. Line 203 (Table 4): Drop the number of complications from this tables; not as important or of any value to the study.

Answer: OK. Done.

32. Line 207: Drop "main" and add "significantly" before "associated".

Answer: OK. Done.

33. Line 215 (Table 5): The reference groups indicated here are mostly not correct. E.g. check Age, PCV, Hib, Vomitting, irritability.

Answer: OK. Done. All reference values have been corrected.

34. Line 215 (Table 5): The percentages presented for the survived and died groups for each row should be the proportions based on row totals and not column totals. E.g. Age <5 survived 129 (82.7%) died (17.3%), Age >=5 survived (74.5%) died (25.5%).

Answer: OK. Done. All the percentages have been changed based on your comments.

35. Line 226 (Table 6): There is no need to present the numbers of patients who survived or died in each row. It might be better to include a column of the Crude OR in this table, as this allows to compare the crude and adjusted at the same instance.

Answer: OK. Done. Numbers of survived and dead have been removed. Also, a new column of Crude OR has been added.

36. Line 238-239: Consider dropping the Canada study, as the age of the study population is not the same

Answer: OK. Removed.

37. Line 240-243: Consider dropping the UK & Ireland, France, and Taiwan study, as the as the age of the study population is not the same.

Answer: OK. Removed.

38. Line 247: Consider dropping the Saudi Arabia study as the age is also not the same

Answer: OK. Removed.

39. Line 252-254: Is there a reference to this statement?

Answer: Sorry, unfortunately “No”. This information is based on the experience of our research team and pediatricians of the hospital. This is a very common problem.

40. Line 270: change from "is declined" to "has declined"

Answer: Ok. Done.

41. Line 282: drop "is" before "may"

Answer: OK. Done.

**Many thanks for your kind comments.**
