# Supplementary material for: Outcome of acute bacterial meningitis among children in Kandahar, Afghanistan: A prospective observational cohort study
Source: PLoS One. 2022 Apr 11;17(4):e0265487. doi: 10.1371/journal.pone.0265487 (PMC9000062; doi:10.1371/journal.pone.0265487)
Supplement: Attachment — Submitted filename: Response to Reviewers.docx [file pone.0265487.s006.docx]

First of all, I am very thankful of the useful comments of the reviewer.

As most of the comments were related to English language grammar, so I sent the manuscript to a native speaker of English. Many thanks to him, as he corrected the whole manuscript grammatically.

Just one thing to remind you that due to thorough grammatical review, the line numbers have changed from the preview version.

Thanks again for your kind and fruitful comments.

Following are the comments, followed by my answers:

6. Review Comments to the Author

Please use the space provided to explain your answers to the questions above. You may also include additional comments for the author, including concerns about dual publication, research ethics, or publication ethics. (Please upload your review as an attachment if it exceeds 20,000 characters)

Reviewer #1: The authors have done a good job in addressing the previous comments and the quality of the manuscript is greatly improved.
There are some minor issues that have been noted at this time and these need to be addressed so as to improve the readability and coherence of the manuscript:
1. Lines 39-42: rewrite this sentence - "Concurrent infections..."; has logical and grammatical errors.

Answer: OK. Now this sentence (as well as the whole manuscript) is reviewed and corrected by a native English speaker.
2. Lines 47-48: consider removing the sentence "The coverage of PCV and Hib...", can be incorporated with the sentence in line 49 - "Increasing vaccine coverage..."

Answer: OK done. Now the sentence "The coverage of PCV and Hib..." is dropped.
3. Line 56: change from "the case fatality" to "a case fatality"

Answer: OK done. It is changed accordingly.
4. Line 57: rewrite to "7% and a risk of neurological..."

Answer: Ok done. Now it is re-written and corrected by a native English speaker.

5. Line 72: use number formatting for the figures presented i.e. 63,001; 16,335; 7,302 and 6,907

Answer: OK done. Now it is corrected as per your advice.

6. Lines 83-84: rewrite the sentence from "Unfortunately, there is currently..." to "Currently, there is no published data on the outcome of ABM in children in Afghanistan and the factors associated with poor outcomes."

Answer: OK done. Now it is re-written and corrected by a native English speaker.

7. Line 85: remove the word "treatment" and also reword "outcome of ABM patients.." to "outcome of ABM in children..."

Answer: OK done. Now corrected as per your advice.

8. Line 91: should read "during a 12-month period"

Answer: OK done.

9. Line 109: Change to Study design and participants, then move the study design and period to be part of this section

Answer: OK done.

10. Lines 92-108: this should be under a section called "Study procedures" and should come after the Study design and participants section

Answer: OK done.

11. Lines 147-148: remove this section altogether; no added value of having this presented in the manuscript.

Answer: OK done.

12. Line 173: Median of 4.8 seems incorrect i.e. median is usually a whole number or half of two whole numbers e.g. 4.5 if the median lies between 4 and 5; please check again how you calculated this, as 4.8 would be more of mean than median.

Answer: Sorry. Thanks for the very good point. Yes, you are right. It is mean (SD). Now I corrected it in the text and table.

12. Line 173: 207 (52.7%) children under age 5, this is discrepant with the data presented in table 1 - please check

Answer: Sorry. Thanks for the very good point. Yes, you are right. Now I corrected it.

13. Line 182: Table 1:
- median (IQR) - present the IQR as a range i.e. the 25th and 75th centile figures rather than the
actual value of the range e.g. for age it would be median (IQR) - 4.5(2.5-7); (please do this
wherever IQR is presented i.e. Table 3)
- consider combining HiB and PCV as one category - since you mentioned in your previous response
that you confirmed everyone who received HiB also received PCV. (also do this for all the other
tables where you present Hib and PCV data i.e. Tables 5 and 6)

Answer: OK thanks. Done. IQR are now presented as a range i.e. the 25th and 75th centile figures. Also, PCV and Hib have been combined as one category.

14. Lines 184 - 185: some of the footnotes not relevant to this table e.g. cm, CSF, mg/dl, kg

Answer: Many thanks for the good point. Now I have removed the irrelevant ones.

15. Line 200: you refer to mean +/- SD while in the table it is median (IQR); please confirm which it is and correct accordingly

Answer: Thanks. Sorry for that. It was median (IQR). Now corrected.

16. Line 236: Table 6 - please re-look at the adjusted OR for purple rash, this seems to be an error which is most likely due to coding; this kind of dramatic reversal cannot be explained by the other variables in the model.

Answer: Thanks for that. Data was re-analyzed. There was in fact an error in coding. Now new corrected values have been written, i.e., COR (95% CI) is 4.0 (1.5-10.6), AOR (95%CI) is 3.1 (0.9-8.3), and the p-value is 0.05.

17. Line 244: drop "living in over-crowded houses, presence of complications" as this was not assessed in the univariate analysis.

Answer: OK. Now "living in over-crowded houses, presence of complications" is dropped.

18. Line 247: "change from in-hospital mortality rate" to "in-hospital case fatality" and also correct spelling of "Compared"

Answer: OK. Now correct as per your advice.

19. Line 255: consider dropping "huge"

Answer: OK. Now “huge” is removed.

20. Lines 259-261: consider dropping this entire sentence: "The in-hospital mortality...better treatment". This is because you have dealt with the self-discharge group in the outcome analysis and the 21.5% fatality was of the group that did not include the self-discharged group.

Answer: OK. Now this sentence is dropped.

21. Line 305: change from "mortality rate" to "case fatality"

Answer: OK. Now changed.

22. Line 307: drop "living in over-crowded houses, presence of complications" as this was not assessed in the univariate analysis.

Answer: OK. Now "living in over-crowded houses, presence of complications" is dropped.

23. Line 313: UNICEF should be in all caps.

Answer: Ok. Now done.

**Many thanks for your kind comments.**
