# Supplementary material for: Outcome of acute bacterial meningitis among children in Kandahar, Afghanistan: A prospective observational cohort study
Source: PLoS One. 2022 Apr 11;17(4):e0265487. doi: 10.1371/journal.pone.0265487 (PMC9000062; doi:10.1371/journal.pone.0265487)
Supplement: Attachment — Submitted filename: Response to Reviewers.docx [file pone.0265487.s007.docx]

First of all, I am very thankful of the useful comments of the reviewer.

Following are the comments, followed by my answers:

**Comment 1:** The major substantive issue remaining is the CSF white count. What lab method was used? If it was the hemocytometer, mononuclear cells are lumped together (rather than a specific reference to lymphocytes). Thus, the percent of neutrophils and mononuclears should add up to 100%. That does not appear to be the case for the data shown in Table 3. It is also highly unusual for acute bacterial meningitis to have a mononuclear predominance as the data in Table 3 suggest; uncommon enough to require re-evaluation of the presumptive diagnosis of bacterial meningitis.

Answer: First of all, thanks a lot for the very good and important comment.

Yes, lab used hemocytometer method for CSF white cells. You are right, they were mononuclear cells (not lymphocytes). Now lymphocytes have been changed to “mononuclear cells”.

To answer second part of your comment, we re-analyzed all the data. Unfortunately, there were mistakes in the data analysis of CSF white counts. We are very sorry for this. The data in Table 3 and text were corrected based on the re-analysis.

Once again, many thanks for the extremely useful comment. Also, accept our apologies for the mistakes.

**Comment 2:** The follow up is not available for the 72 who “discharged themselves” from the hospital. I assume that means that the parents removed them against medical advice. Is anything known about their outcome? Especially, how many were removed because the parents thought they were dying?

Answer: These 72 children were discharged by their parents against medical advice. As their parents did not ask us before discharge (also, they did not tell us the reason for the self-discharge), so we could not follow them up and did not know about their outcome. In conclusion, we do not know the exact reason of their self-discharge. We know just one thing that most of these 72 LAMA patients were in critical situation and possibly their parents thought they were dying and took their children to Kabul or another country (especially Pakistan or India) for better treatment.

**Line 30:** “of” after total is missing. Also, median has been used instead of mean

Answer: OK. Done. “of” is added while median is changed to mean.

**Line 58:** a citation is needed at the end of the first sentence

Answer: OK. Citation is added.

**Line 156:** the 0C did not come through correctly on my version of the pdf

Answer: OK. Done. Degree centigrade symbol is corrected.

**Line 158:** eliminate the space before the comma

Answer: OK. Done. Space is removed.

**Many thanks for your kind and useful comments.**
