# Supplementary material for: Outcome of acute bacterial meningitis among children in Kandahar, Afghanistan: A prospective observational cohort study
Source: PLoS One. 2022 Apr 11;17(4):e0265487. doi: 10.1371/journal.pone.0265487 (PMC9000062; doi:10.1371/journal.pone.0265487)
Supplement: Attachment — Submitted filename: Response to Reviewers.docx [file pone.0265487.s008.docx]

First of all, I am very thankful of the useful comments of the reviewer.

Following are the comments, followed by my answers:

Thank you for your revision. There are still two issues that should be covered before publication:

1. The fact that 72 of the children were critical ill when brought home by their parents means that the mortality rate may have been much higher than the stated 21.5%. If all of these died, the mortality would have actually been 35.9%. It would be appropriate to raise this possibility at the beginning of the discussion where the mortality rate is discussed.

Answer: OK. Done. This important comment is added to the discussion.

2. I am surprised by the high percent of CSFs with a mononuclear predominance (25%). Is it possible that some of these were actually tuberculous meningitis? That possibility should also be raised in the discussion.

Answer: OK. Done. The possibility of some TBM patients has been raised in the discussion part of the manuscript.

**Many thanks for your kind and useful comments.**
